# Immunomodulatory Mechanisms Underlying Neurological Manifestations in Long COVID: Implications for Immune-Mediated Neurodegeneration

**DOI:** 10.3390/ijms26136214

**Published:** 2025-06-27

**Authors:** Zaw Myo Hein, Thazin, Suresh Kumar, Muhammad Danial Che Ramli, Che Mohd Nasril Che Mohd Nassir

**Affiliations:** 1 Department of Basic Medical Sciences, College of Medicine, Ajman University, Ajman P.O. Box 346, United Arab Emirates; z.hein@ajman.ac.ae; 2 Faculty of Medicine, Nursing and Health Sciences, SEGi University, Petaling Jaya 47810, Selangor, Malaysia; thazin@segi.edu.my; 3 Department of Diagnostic and Allied Health Science, Faculty Health and Life Sciences, Management and Science University, Shah Alam 40100, Selangor, Malaysia; sureshkumar@msu.edu.my; 4 Department of Anatomy and Physiology, Faculty of Medicine, Universiti Sultan Zainal Abidin, Kuala Terengganu 20400, Terengganu, Malaysia

**Keywords:** COVID-19, SARS-CoV-2, neurological disease, immune system, long COVID-19

## Abstract

The COVID-19 pandemic has revealed the profound and lasting impact of severe acute respiratory syndrome coronavirus 2 (SARS-CoV-2) on the nervous system. Beyond acute infection, SARS-CoV-2 acts as a potent immunomodulatory agent, disrupting immune homeostasis and contributing to persistent inflammation, autoimmunity, and neurodegeneration. Long COVID, or post-acute sequelae of SARS-CoV-2 infection (PASC), is characterized by a spectrum of neurological symptoms, including cognitive dysfunction, fatigue, neuropathy, and mood disturbances. These are linked to immune dysregulation involving cytokine imbalance, blood–brain barrier (BBB) disruption, glial activation, and T-cell exhaustion. Key biomarkers such as interleukin-6 (IL-6), tumor necrosis factor-alpha (TNF-α), glial fibrillary acidic protein (GFAP), and neurofilament light chain (NFL) correlate with disease severity and chronicity. This narrative review examines the immunopathological mechanisms underpinning the neurological sequelae of long COVID, focusing on neuroinflammation, endothelial dysfunction, and molecular mimicry. We also assess the role of viral variants in shaping neuroimmune outcomes and explore emerging diagnostic and therapeutic strategies, including biomarker-guided and immune-targeted interventions. By delineating how SARS-CoV-2 reshapes neuroimmune interactions, this review aims to support the development of precision-based diagnostics and targeted therapies for long COVID-related neurological dysfunction. Emerging approaches include immune-modulatory agents (e.g., anti-IL-6), neuroprotective drugs, and strategies for repurposing antiviral or anti-inflammatory compounds in neuro-COVID. Given the high prevalence of comorbidities, personalized therapies guided by biomarkers and patient-specific immune profiles may be essential. Advancements in vaccine technologies and targeted biologics may also hold promise for prevention and disease modification. Finally, continued interdisciplinary research is needed to clarify the complex virus–immune–brain axis in long COVID and inform effective clinical management.

## 1. Introduction

The worldwide impact of the COVID-19 pandemic has surpassed its initial characterization as a respiratory illness, revealing a multifaceted viral syndrome with systemic implications. Among the most complex and debilitating consequences of acute respiratory syndrome coronavirus 2 (SARS-CoV-2) infection are its persistent effects on the nervous system. Individuals recovering from the acute phase of COVID-19 often experience prolonged neurological symptoms, including cognitive impairment (“brain fog”), fatigue, headaches, paresthesia, and dysautonomia, which collectively fall under the umbrella of post-acute sequelae of SARS-CoV-2 infection (PASC), also known as long COVID [1,2].

Recognizing the need for a standardized clinical framework, the World Health Organization (WHO) established a case definition for the post-COVID-19 condition in October 2021. According to this definition, the condition occurs in individuals with a history of probable or confirmed SARS-CoV-2 infection, typically three months from the onset of COVID-19, with symptoms lasting for at least two months and not attributable to alternative diagnoses. Common symptoms include fatigue, shortness of breath, and cognitive dysfunction, which can impact daily functioning [3]. Recent systematic reviews and meta-analyses have sought to quantify the prevalence of long COVID symptoms. A study by Mudgal et al. [4] reported that approximately 45.9% of COVID-19 survivors experienced at least one persistent symptom 90 days post-infection. Furthermore, neurological manifestations such as cognitive impairment, headaches, and mood disorders have been frequently documented.

One of the most striking features of SARS-CoV-2 is its profound capacity to modulate the human immune system [5]. This virus interacts with host immunity at multiple levels, inducing a cascade of immune dysfunctions ranging from cytokine storms in the acute phase to chronic [6], low-grade neuroinflammation in the post-acute phase [7]. SARS-CoV-2 hijacks immune signaling pathways, perturbs cytokine networks, and disrupts immune cell function, alterations that have downstream effects on both central and peripheral nervous systems [8,9]. Several mechanisms underlie the neurological manifestations of long COVID, many of which are immunologically driven. These include (i) direct viral neuro-invasion, (ii) immune-mediated damage via pro-inflammatory cytokines (e.g., IL-6 and TNF-α), (iii) the breakdown of the BBB, (iv) glial cell activation, (v) molecular mimicry triggering autoimmunity, and (vi) endothelial dysfunction leading to cerebral microvascular injury [10,11]. Importantly, persistent elevations of neuroimmune biomarkers such as GFAP, NFL, C-C motif ligand 2 (CCL2) (or monocytic chemotactic protein 1, MCP-1), and galectin-9 serve as evidence of ongoing immune activation within the central nervous system (CNS) [12].

Thus, understanding how SARS-CoV-2 alters immune responses, particularly in the context of neurodegeneration, offers critical insights into the long-term neurological burden of COVID-19. This is especially relevant for individuals with pre-existing immune dysfunction, such as those with autoimmune conditions, cancer, or advanced age, who appear to be disproportionately affected. In this narrative review, we examine the immunological mechanisms that drive neurological symptoms in long COVID, discuss the molecular and cellular pathways involved, and evaluate potential diagnostic and therapeutic targets rooted in immune modulation. Our goal is to provide an immunology-focused framework for interpreting the neurological sequelae of SARS-CoV-2 infection, thereby aligning with the broader goals of the post-pandemic reflection on how viruses, particularly SARS-CoV-2, reshape human immunology and health.

### Literature Search Strategies

This narrative review primarily includes peer-reviewed articles published between January 2020 and April 2025, sourced from databases including PubMed, Scopus, and Web of Science. In total, over 140 references were cited, encompassing clinical cohort studies, postmortem analyses, in vitro and in vivo experimental models, and reviews focusing on SARS-CoV-2-induced neuroimmune dysfunction. Emphasis was placed on studies reporting the neurological sequelae of long COVID, variant-specific neuropathogenesis, and potential immunotherapeutic strategies.

## 2. Immunopathogenesis of Neuro-COVID: Molecular Mechanisms of Immune-Driven Neural Injury

The neurological sequelae of COVID-19, both in its acute and post-acute (long COVID) phases, are increasingly understood to stem predominantly from immune-mediated processes rather than direct viral neuroinvasion. Although SARS-CoV-2 ribonucleic acid (RNA) and proteins have been detected in brain tissues and cerebrospinal fluid (CSF) in selected cases [13], compelling evidence now supports that the majority of neuro-COVID manifestations result from systemic inflammation, aberrant immune responses, and subsequent neuroimmune dysregulation. These processes involve a complex interplay between cytokine-induced neuroinflammation, the disruption of the BBB, autoimmunity via molecular mimicry, persistent glial activation, and microvascular endothelial injury.

### 2.1. Cytokine Storm and Neuroinflammation

A defining feature of severe COVID-19 is the cytokine storm, a hyperinflammatory state characterized by the excessive release of interleukins (e.g., IL-6 and IL-1β), TNF-α, interferon-γ (IFN-γ), and chemokines (e.g., CCL2 and CXCL10) [14,15]. This systemic immune activation not only promotes multiorgan damage but also exerts profound effects on the CNS. Elevated pro-inflammatory cytokines increase BBB permeability, allowing the infiltration of peripheral immune cells and serum proteins into the CNS, which in turn activates microglia and astrocytes [16]. Moreover, computational studies have predicted that key neuroinflammatory pathways, such as nuclear factor kappa beta (NF-κB) and Janus kinase/signal transducer and activator of transcription (JAK/STAT), may be regulated by microRNAs involved in COVID-19-related immune responses [17]. While experimental validation in CNS-resident immune cells remains limited, these findings provide valuable insights into potential regulatory mechanisms driving neuroinflammation.

Furthermore, longitudinal studies have demonstrated that elevated IL-6 and TNF-α levels correlate with cognitive dysfunction, fatigue, and mood disorders in long COVID, suggesting that persistent low-grade inflammation contributes to post-viral neurocognitive syndromes [18,19]. These findings align with positron emission tomography (PET) imaging data showing widespread glial activation and hypometabolism in the frontal and temporal brain regions in long COVID patients [20].

### 2.2. BBB Disruption

The BBB is a highly selective barrier that maintains CNS homeostasis and protects the brain from peripheral immune surveillance. Inflammatory cytokines, oxidative stress, and endothelial injury during COVID-19 weaken tight junction proteins such as claudin-5 and occludin, leading to increased BBB permeability [21]. This compromise allows immune mediators, cytokines, and potentially viral components to enter the CNS and exacerbate neuroinflammation.

Recent studies have identified elevated levels of S100B and matrix metalloproteinases (MMP-9 and MMP-2) in the serum and CSF of COVID-19 patients as biomarkers of BBB disruption [22,23]. Moreover, experimental studies have confirmed that cerebrovascular endothelial cells express angiotensin-converting enzyme 2 (ACE2), the SARS-CoV-2 entry receptor, making them directly susceptible to viral spike protein-mediated damage, even in the absence of full viral replication [24,25,26,27]. This endothelial involvement promotes a pro-thrombotic and inflammatory milieu, perpetuating CNS injury.

### 2.3. Autoimmunity and Molecular Mimicry

Emerging evidence suggests that SARS-CoV-2 can trigger autoimmune responses through molecular mimicry whereby the virus structure resembles that of a host molecule [28]. Epitope spreading or the immune system expands its targeting of epitopes beyond the initial, specific target and induces bystander activation which is the activation of T cells (specifically CD8+ T cells) that are not directly targeted by a pathogen or antigen [29]. Moreover, viral proteins such as the spike (S) and nucleocapsid (N) proteins share sequence homology with several human proteins, including neuronal antigens. This structural resemblance may lead to cross-reactive T cells and autoantibody production [30,31].

Autoimmune neurological syndromes reported post-COVID-19 include Guillain–Barré syndrome (GBS), acute disseminated encephalomyelitis (ADEM), autoimmune encephalitis, and transverse myelitis [32,33,34,35,36]. In several cases, anti-ganglioside and anti-N-methyl-D-aspartate receptor (NMDAR) antibodies were detected in CSF, even in patients without prior autoimmune predisposition [37,38]. Several meta-analyses reported a significantly increased incidence of autoimmune neurological disorders following SARS-CoV-2 infection compared to non-infected controls, with GBS being the most common manifestation [39,40].

### 2.4. Persistent Glial Activation and Neuroimmune Reprogramming

Postmortem histopathological analyses and advanced neuroimaging have revealed the sustained activation of microglia and astrocytes in brain regions including the hippocampus, basal ganglia, and brainstem, often in the absence of detectable viral RNA [41]. This raises an important mechanistic question: whether glial activation is a residual effect of prior viral neuroinvasion that persists after viral clearance or arises independently due to peripheral immune activation and cytokine signaling. Both scenarios are plausible and are supported by emerging evidence. For instance, even in the absence of detectable viral presence in the CNS, elevated levels of systemic IL-6, TNF-α, and MCP-1 have been shown to cross a compromised blood–brain barrier (BBB) and induce neuroinflammation through microglial priming [42]. Conversely, in a subset of animal models with confirmed CNS viral presence, glial activation may represent a direct innate immune response to viral antigens [43,44]. These distinct pathways, virus-dependent vs. virus-independent, carry implications for therapeutic targeting, with antiviral strategies being more relevant in the former and immunomodulatory approaches favored in the latter. Glial activation contributes to synaptic dysfunction, excitotoxicity via excessive glutamate release, oxidative stress, and neuronal apoptosis [45]. Long COVID patients often display persistent symptoms such as “brain fog,” impaired memory, and sleep disturbances, which may reflect glial-driven dysregulation of synaptic plasticity and circadian homeostasis [1]. Additionally, recent studies have shown that SARS-CoV-2 infection leads to epigenetic reprogramming of glial cells, prolonging their pro-inflammatory phenotype even after systemic recovery [46,47].

### 2.5. Endothelial Dysfunction and Microvascular Injury

COVID-19 is increasingly recognized as a vascular disease with prominent endothelial dysfunction. SARS-CoV-2 induces endothelial activation via direct viral interactions and indirectly through immune-mediated mechanisms. Elevated levels of IL-6, vascular endothelial growth factor (VEGF), and von Willebrand factor promote endothelial inflammation, increase vascular permeability, and facilitate microthrombus formation.

Neuroimaging studies have revealed cerebral microbleeds, lacunar infarcts, and white matter hyperintensities in COVID-19 survivors, which are indicative of cerebral small vessel disease (CSVD) and microvascular pathology [48,49]. These changes are associated with neurocognitive decline and depression in long COVID. Importantly, endothelial dysfunction may persist for months after infection, driven by persistent low-grade inflammation, mitochondrial dysfunction, and immune dysregulation, contributing to ongoing neurovascular compromise.

**Figure 1:**
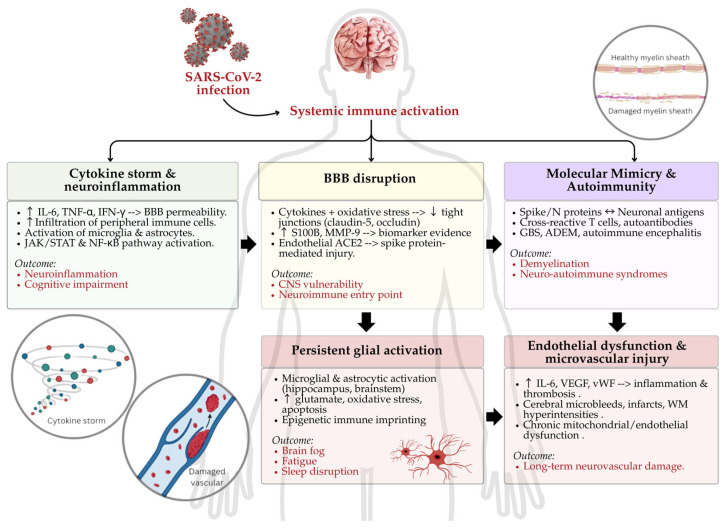
Schematic illustration of proposed mechanisms of neuroimmune dysfunction following SARS-CoV-2 infection. Acute respiratory syndrome coronavirus 2 (SARS-CoV-2) may trigger neuroimmune impairment through five interconnected pathways: (1) A cytokine storm increases blood–brain barrier (BBB) permeability and promotes neuroinflammation; (2) BBB disruption via oxidative stress and spike-mediated endothelial injury facilitates central nervous system (CNS) entry; (3) molecular mimicry leads to autoimmune syndromes such as Guillain–Barré syndrome (GBS) and acute disseminated encephalomyelitis (ADEM); (4) persistent glial activation contributes to brain fog, fatigue, and sleep disturbance; and (5) endothelial dysfunction causes microvascular injury and long-term neurovascular damage. IL-6, interleukin-6; TNF-α, tumor necrosis factor-alpha; IFN-γ, interferon-gamma; JAK/STAT, Janus kinase/signal transducer and activator of transcription; NF-κB, nuclear factor kappa-light-chain-enhancer of activated B cells; S100B, S100 calcium-binding protein B, MMP-9, matrix metalloproteinase-9; ACE2, angiotensin-converting enzyme 2; VEGF, vascular endothelial growth factor; vWF, von Willebrand factor; WM, white matter. Different colorful dots in the cytokine storm figure represent different pro-inflammatory cytokines. The upward arrow (↑) represents increased, the downward arrow (↓) represents decreased/reduced, and ↔ represents similar/indicating.

In summary, emerging evidence suggests that SARS-CoV-2 can trigger a cascade of neuroimmune mechanisms that contribute to long-term neurological sequelae. Figure 1 illustrates the proposed pathophysiological pathways linking systemic immune activation to CNS dysfunction in long COVID.

## 3. Neurological Sequelae of Long COVID: Clinical Presentation and Immunological Correlates

Long COVID presents a broad spectrum of neurological manifestations, which range from mild cognitive impairment to serious neuroimmune complications. While these symptoms may initially appear heterogeneous, accumulating evidence indicates that they share common immunopathological underpinnings. Persistent neuroinflammation, immune cell dysregulation, and cytokine-mediated damage are central to these long-term effects.

### 3.1. Cognitive Impairment and Brain Fog

Cognitive impairment, colloquially termed “brain fog”, is among the most frequently reported symptoms of long COVID, affecting a significant proportion of patients across all age groups, including those with mild initial infections [50]. These cognitive disturbances often manifest as deficits in short-term memory, attention, processing speed, and executive functioning. Functional MRI studies have demonstrated hypometabolism in the frontoparietal and limbic regions, suggesting region-specific dysfunction that correlates with clinical symptoms [51,52].

A growing body of evidence links this cognitive phenotype with biomarkers indicative of neural injury and inflammation. Elevated concentrations of NFL and GFAP in both serum and CSF have been documented in patients exhibiting cognitive symptoms weeks to months after acute SARS-CoV-2 infection [12]. NFL is a marker of axonal degeneration, while GFAP reflects astrocytic activation or injury, and their persistence implies ongoing neurodegeneration or glial reactivity. Immunologically, brain fog in long COVID is associated with a sustained elevation of pro-inflammatory cytokines, particularly interleukin-6 (IL-6), TNF-α, and CCL2/MCP-1. These mediators are known to compromise BBB integrity, recruit monocytes into the CNS, and induce neurotoxic astrocytic phenotypes [53]. Chronic exposure to such an inflammatory milieu may impair hippocampal function and disrupt synaptic plasticity, key processes underlying memory and executive function.

Recent studies provide new insights into potential mechanisms underlying cognitive sequelae in long COVID. (1) Peripheral immune cell reprogramming: Longitudinal single-cell analyses of peripheral blood from long COVID patients have revealed the expansion of non-classical monocytes and exhaustion signatures in CD4+ T cells, which may impair immune surveillance and facilitate chronic inflammation [54]. (2) Mitochondrial dysfunction: Emerging metabolomic data indicate disrupted energy metabolism in neurons due to impaired mitochondrial function, possibly secondary to cytokine-mediated oxidative stress [55]. This mitochondrial injury could underlie the cognitive fatigue often reported in long COVID. (3) Autoantibodies: A subset of long COVID patients exhibits elevated levels of autoantibodies against neuronal and glial antigens, e.g., anti-myelin oligodendrocyte glycoprotein (MOG) and anti-NMDA receptor, suggesting that autoimmunity may play a role in sustaining CNS inflammation and cognitive decline [56,57]. (4) Gut–brain axis disruption: Alterations in gut microbiota composition, particularly the depletion of short-chain fatty acid (SCFA)-producing taxa, have been linked to worsened neurocognitive outcomes, potentially via systemic inflammation and impaired microglial regulation [58]. This offers a novel therapeutic angle via microbiome modulation.

Understanding the immunological underpinnings of cognitive impairment in long COVID has substantial implications. First, the identification of reliable biomarkers (e.g., NFL, GFAP, and IL-6) could facilitate early diagnosis and the stratification of patients at risk for persistent neurological dysfunction. Second, therapeutic strategies targeting glial activation, mitochondrial repair, or cytokine neutralization (e.g., anti-IL-6 therapy) warrant investigation in clinical trials. Finally, given the intersection of long COVID with neurodegenerative processes, there is an urgent need to monitor affected individuals longitudinally for increased risk of conditions such as Alzheimer’s disease or Parkinsonian syndromes.

### 3.2. Neuropathy and Paresthesia

Peripheral neuropathies, including sensory disturbances such as numbness, tingling, burning pain, and allodynia, have been increasingly documented in long COVID cohorts, often emerging weeks after respiratory symptoms resolve [59]. These manifestations predominantly reflect small fiber neuropathy (SFN) and may involve both somatic and autonomic fibers [60,61]. While the pathophysiology remains incompletely defined, immune-mediated mechanisms appear central. Elevated levels of pro-inflammatory cytokines such as IL-8 and TNF-α promote leukocyte trafficking and facilitate neurotoxic environments in dorsal root ganglia and peripheral nerves [59].

Notably, skin biopsy studies in long COVID patients have demonstrated reduced intra-epidermal nerve fiber density, a hallmark of SFN, correlating with increased circulating complement components and anti-neuronal autoantibodies [62]. Electrophysiological assessments further support demyelination and axonal damage, consistent with both immune-mediated and metabolic disruption of nerve integrity [63]. In addition, recent single-cell immune profiling of patients with post-viral neuropathy has revealed persistent activation of CD9+ monocytes and pro-inflammatory macrophages in peripheral nerve tissues, implicating a chronic inflammatory state as a driver of neuropathic symptoms [64]. These findings support immunomodulatory interventions as a potential therapeutic strategy.

### 3.3. Mood Disorders and Neuropsychiatric Symptoms

Neuropsychiatric complications, including depression, anxiety, insomnia, and post-traumatic stress disorder (PTSD), are widely reported in individuals with long COVID and often co-occur with cognitive deficits and fatigue [65]. These disturbances are increasingly understood as outcomes of neuroimmune dysregulation, rather than mere psychological reactions to illness.

Cytokines such as IL-1β, IL-6, and IFN-γ exert profound effects on the CNS by modulating neurotransmitter synthesis (e.g., reducing serotonin via tryptophan degradation), altering synaptic plasticity, and disrupting neurogenesis [66]. Elevated C-reactive protein (CRP) and soluble IL-6 receptor levels have been consistently associated with depression severity, particularly in patients with pre-existing vulnerability to affective disorders [67]. Additionally, dysregulation of the hypothalamic–pituitary–adrenal (HPA) axis has been implicated, with many long COVID patients demonstrating elevated cortisol and acetylcholine levels, alongside impaired negative feedback inhibition [68]. Such endocrine dysfunction further amplifies systemic inflammation and can perpetuate a cycle of neuropsychiatric distress. Neuroimaging studies lend further support that the reductions in hippocampal volume and altered connectivity in the default mode network (DMN) have been observed in long COVID patients with mood disturbances, mirroring findings seen in primary psychiatric conditions [69,70].

### 3.4. Postural Orthostatic Tachycardia Syndrome (POTS)

Postural orthostatic tachycardia syndrome (POTS) is a prominent form of dysautonomia emerging in the aftermath of SARS-CoV-2 infection. Defined by a sustained heart rate increase of ≥30 beats per minute upon standing (≥40 bpm in adolescents), POTS is frequently accompanied by fatigue, palpitations, light-headedness, and cognitive dysfunction (“brain fog”) [71,72].

Pathophysiologically, post-COVID POTS is hypothesized to arise from autoimmune dysregulation of autonomic ganglia and baroreceptor pathways. Elevated titers of autoantibodies targeting adrenergic (β1/β2) and muscarinic (M2/M4) receptors have been reported in affected patients, suggesting functional disruption of sympathetic and parasympathetic signaling [73,74]. Concurrently, increased levels of IL-6, CCL2/MCP-1, and soluble CD30 support a chronic inflammatory microenvironment that further impairs autonomic function [75,76]. Moreover, recent findings from tilt-table testing cohorts demonstrate aberrant vascular reactivity and cerebral hypoperfusion, suggesting an interplay between vascular endothelial dysfunction and neuroimmune signaling. Some researchers have drawn parallels between post-COVID POTS and autoimmune autonomic ganglionopathy, reinforcing the role of targeted immune therapies such as intravenous immunoglobulin, steroids, or β-blockers in select cases [77,78].

### 3.5. Neurodegenerative Syndromes: Parkinsonism and Dementia

An unsettling trend emerging in the long COVID literature is the increased incidence and progression of neurodegenerative diseases such as Parkinson’s disease (PD) and Alzheimer’s disease (AD). While causality remains under investigation, several converging mechanisms are being implicated. SARS-CoV-2 infection induces chronic microglial activation and mitochondrial dysfunction, which are central to neurodegenerative cascades. Elevated levels of α-synuclein, total tau, phosphorylated tau (pTau), and amyloid beta 42 (Aβ42) have been detected in the CSF and serum of long COVID patients with progressive cognitive or motor symptoms, suggesting a possible acceleration of underlying neurodegenerative processes [79].

Neuroinflammatory cytokines, including IL-1β, IL-10, and soluble TNF receptors (sTNFR1/2), are persistently elevated in these individuals, reflecting a pro-inflammatory CNS milieu that may promote amyloidogenesis and α-synuclein aggregation [80]. Research indicates that SARS-CoV-2 can infect human pluripotent stem cell-derived dopaminergic neurons, leading to cellular senescence and reduced numbers of neuromelanin-positive and tyrosine hydroxylase-positive neurons in the substantia nigra of COVID-19 patients. These findings suggest that SARS-CoV-2 infection may contribute to dopaminergic neuron loss, mimicking PD pathology [81]. Additionally, studies have observed morphological changes, increased apoptosis, and decreased neurogenesis in the hippocampus of COVID-19 patients, indicating synapse degeneration similar to that seen in AD [82].

Case reports have documented patients developing Parkinsonism following SARS-CoV-2 infection, presenting with symptoms like rigidity and bradykinesia. Functional imaging using [18F]-DOPA PET and DaT-SPECT has demonstrated decreased dopamine uptake in the striatum, indicating nigrostriatal dysfunction typical of PD [83,84]. Importantly, emerging studies also suggest that individuals with pre-existing or prodromal neurodegenerative disorders may be at heightened risk of deterioration following SARS-CoV-2 infection. Patients with PD have shown increased rates of motor symptom worsening, dysautonomia, and even akinetic crises during or after COVID-19, possibly due to systemic inflammation, dopaminergic stress, or altered medication pharmacokinetics [85,86]. Similarly, individuals with AD or mild cognitive impairment may experience accelerated cognitive decline, neuropsychiatric symptom flare-ups, or functional regression [87,88]. These outcomes may reflect synergistic effects between chronic neurodegeneration and COVID-19-induced glial activation, BBB disruption, or cytokine-induced synaptic dysfunction. Recent cohort data from nursing homes and memory clinics also support this risk amplification, highlighting the vulnerability of neurodegenerative populations to SARS-CoV-2-mediated exacerbation of CNS pathology [89,90]. This underscores the importance of post-infection monitoring and tailored neuroprotective strategies in high-risk populations.

### 3.6. Autoimmune Neurological Disorders

SARS-CoV-2 infection has been increasingly linked to the emergence or exacerbation of autoimmune neurological syndromes, including GBS, autoimmune encephalitis, transverse myelitis, multiple sclerosis (MS)-like relapses, and systemic lupus erythematosus (SLE)-related CNS flares [91,92,93]. These disorders often manifest within weeks of infection and reflect molecular mimicry between viral antigens and neural epitopes, triggering aberrant T- and B-cell activation.

Elevated levels of neuronal autoantibodies such as anti-glutamic acid decarboxylase 65 (anti-GAD65), anti-NMDA receptor, and anti-AQP4 have been reported in both the serum and CSF of affected individuals, often accompanied by intrathecal oligoclonal bands and complement activation [94,95,96]. These markers suggest a breach of immune tolerance and BBB dysfunction, enabling pathogenic immune cells and antibodies to access CNS compartments. In GBS, ganglioside-targeting autoantibodies (e.g., anti-GM1) have been detected, while in autoimmune encephalitis, cytokine signatures rich in IL-12, IL-18, IFN-γ, and CXCL10 point to Th1-skewed responses as key drivers of neuroinflammation [97,98].

Overall, there is an urgent need to better delineate the post-infectious versus true autoimmune phenotypes of these conditions, as the therapeutic implications differ significantly. To distinguish post-infectious sequelae from true autoimmune neuroimmune syndromes, it is essential to examine their associated immune signatures. Table 1 provides an overview of key immune mediators and biomarkers implicated in various post-COVID-19 neurological manifestations.

## 4. Variant-Specific Neuropathogenesis: Immune Signatures and Neurovirulence of SARS-CoV-2 Strains

While the previous sections focused on immunological mechanisms and clinical sequelae broadly observed across COVID-19 cases, it is important to consider that the initiating features of SARS-CoV-2 infection including viral entry routes, neurotropism, and variant-specific immune modulation shape the trajectory and severity of these responses. Since its emergence, SARS-CoV-2 has accumulated multiple mutations, particularly in the spike (S) protein, nucleocapsid, and non-structural proteins, that have not only affected transmissibility and immune evasion but also neuropathogenic potential. Variants of concern (VOCs), such as Alpha, Beta, Delta, Omicron, and their sub-lineages, exhibit distinct neuroinvasive, neurotropic, and neuroinflammatory profiles. These differences likely result from a combination of viral factors (e.g., receptor affinity and fusion kinetics), host immune responses (e.g., cytokine storm vs. immune suppression), and tissue-specific expression of ACE2, transmembrane protease, serine 2 (TMPRSS2), and neuropilin-1. Increasing evidence supports the idea that variant-specific mutations influence both direct viral neuroinvasion and indirect neuroimmune consequences, such as BBB permeability, microglial activation, and systemic cytokine diffusion into the CNS.

### 4.1. D614G Mutation: Elevated Neurovirulence and Inflammatory Reactivity

The D614G mutation, located in the spike protein’s S1 domain, was among the first mutations associated with increased viral fitness and global dominance. Although it did not confer significant immune escape, it enhanced S protein stability and ACE2 binding, facilitating viral entry into diverse cell types, including neurons and glial cells. In both animal models and organoid studies, D614G-infected brains showed (1) enhanced olfactory neuroepithelium invasion, followed by transsynaptic spread to the olfactory bulb and cortex [99]; (2) the induction of interferon-stimulated genes and the upregulation of CXCL10, IL-6, and TNF-α, suggesting robust innate immune activation [100]; and (3) the activation of microglia and astrocytes, as evidenced by elevated Iba1 and GFAP expression [101]. This inflammatory milieu likely contributes to persistent anosmia, cognitive dysfunction, and post-infectious encephalitis, which were more common during early waves of the pandemic. Notably, long COVID patients infected during the D614G wave show higher levels of NFL and GFAP, suggesting long-term CNS injury [102].

### 4.2. Delta Variant: High CNS Entry with Moderate Immune Activation

The Delta (B.1.617.2) variant exhibited high neuroinvasive efficiency but a more restrained pro-inflammatory cytokine profile compared to D614G. Its spike mutations (e.g., L452R and T478K) increased fusogenicity and enhanced cell-to-cell viral spread, possibly aiding CNS penetration via the olfactory mucosa and trigeminal nerve [103,104]. However, in vitro studies utilizing co-culture models of human brain microvascular endothelial cells and glial cells have demonstrated that exposure to the Delta variant’s spike protein induces a moderate inflammatory response. Specifically, there is differential activation of the IL-6 signaling pathway compared to earlier variants, with less pronounced downstream activation of microglial cells [105]. This suggests a variant-specific modulation of the neurovascular unit’s inflammatory response.

Nevertheless, clinically, patients infected with the Delta variant have reported neurological symptoms such as encephalopathy, anosmia (loss of smell), and paresthesia (abnormal sensations). While the exact mechanisms remain under investigation, these symptoms may be associated with the virus’s ability to persist in CNS-associated cells, including pericytes and astrocytes. Notably, studies have identified the SARS-CoV-2 spike protein in astrocytes within the brain tissue of COVID-19 patients, indicating potential viral persistence in these glial cells [106]. Animal studies have provided insights into the Delta variant’s impact on the neurovascular unit. In K18-hACE2 transgenic mice and Syrian hamsters, SARS-CoV-2 infection has been shown to affect brain vascular endothelial cells, leading to increased vascular permeability. This is evidenced by the presence of viral RNA in the brain and associated vascular damage, including perivascular inflammatory cell infiltration and basement membrane disruption, without significant alterations of tight junction proteins [107].

These findings collectively suggest that, despite inducing a comparatively moderate inflammatory response in vitro, the Delta variant can still lead to significant neurological symptoms in patients. The potential for viral persistence in CNS-associated cells and the observed vascular endothelial damage in animal models underscore the importance of monitoring and addressing neurological complications associated with SARS-CoV-2 variants.

### 4.3. Omicron Variant: Altered Neuroinflammatory Profile

Initial reports downplayed the neurotropism of Omicron (BA.1, BA.2, BA.5, and XBB) sub-lineages due to their reduced replication efficiency in lower airway and olfactory epithelial cells. However, emerging evidence suggests that indirect neuroinflammatory effects remain significant. Omicron-infected brain organoids show limited direct neuronal infection but demonstrate marked astrocyte and pericyte stress, which is associated with the upregulation of heat shock proteins, oxidative stress markers, and pro-inflammatory cytokines such as IL-6 and CXCL8 [108].

Studies in mouse models expressing human ACE2 show that Omicron variants can still disrupt BBB integrity through non-structural protein interactions with endothelial junction proteins (e.g., claudin-5 and occludin) [109]. Recent studies have demonstrated that exposing microglia-like cells to the SARS-CoV-2 spike protein, including that from the Omicron variant, activates the NLRP3 inflammasome pathway. This activation occurs even in the absence of viral replication, indicating a non-cytolytic yet highly inflammatory response. Specifically, research utilizing human monocyte-derived microglia (MDMi) has shown that the SARS-CoV-2 spike protein can bind to ACE2 receptors on microglia, leading to the activation of the NLRP3 inflammasome. This process results in the secretion of pro-inflammatory cytokines such as interleukin-1β (IL-1β), contributing to neuroinflammation. Notably, this activation can occur without the need for additional priming signals, highlighting the potent inflammatory potential of the spike protein alone [110]. These findings align with clinical reports of fatigue, cognitive impairment, and dysautonomia, even in patients with “mild” Omicron infections, suggesting that systemic inflammation and glial reactivity may drive symptoms without overt neuroinvasion [19].

### 4.4. Beta and Alpha Variants: Reduced CNS Penetrance and Lower Inflammatory Impact

The Alpha (B.1.1.7) and Beta (B.1.351) variants demonstrated relatively low CNS infectivity in organoid and rodent models, correlating with fewer reported cases of overt neurological syndromes (e.g., seizures and encephalitis) [109]. Despite having mutations (N501Y and E484K) that enhanced spike-ACE2 binding, these variants induced less endothelial and astroglia disruption [109].

Experimental data showed minimal disruption of tight junction proteins and limited upregulation of pro-inflammatory cytokines such as IL-12 and IFN-γ [111]. Nevertheless, both Alpha and Beta could bind to ACE2+ glial cells and TMPRSS2+ endothelial cells, and in rare cases, trigger autoimmune encephalitis or vascular complications (e.g., microthrombosis, vasculitis) [112,113]. Their lower immunogenicity in CNS tissue may reflect a reduced capacity to trigger long-lasting neuroinflammation, though chronic fatigue and mood disorders were still commonly reported, likely mediated through peripheral immune–brain signaling rather than direct neuro-invasion [109].

Given the evolving nature of SARS-CoV-2 and its variants, understanding the differential impact on the nervous system is critical. Table 2 summarizes the variant-specific neuropathogenic mechanisms, immune responses, CNS tropism, and associated clinical features.

While the neuroinvasive potential of SARS-CoV-2 has declined in later variants like Omicron, neurological symptoms remain prevalent, suggesting a paradigm shift from direct viral neurotropism to immune-mediated neurotoxicity. This highlights the importance of monitoring long-term CNS effects not only during acute infection but also in the post-viral period. Emerging technologies such as spatial transcriptomics, single-nucleus RNA-seq, and BBB-on-a-chip platforms will be essential for dissecting variant-specific cellular responses in the brain. Moreover, personalized immunophenotyping may identify vulnerable individuals at risk for persistent neuroimmune activation following infection by specific variants.

## 5. Diagnostic and Biomarker-Based Assessment of Neuroimmune Dysfunction in Long COVID

The diagnosis of neurological complications in long COVID remains complex and often delayed due to several overlapping challenges. These include the absence of standardized diagnostic definitions, the variability in timing and presentation of symptoms, and frequent overlaps with preexisting neurological or psychiatric conditions [114]. Nevertheless, mounting evidence indicates that specific immune and neural biomarkers can offer valuable insights into the pathophysiology of long COVID, especially in relation to neuroimmune dysregulation. These biomarkers help establish a mechanistic link between SARS-CoV-2-induced immune alterations and persistent neurological symptoms, with increasing promise as diagnostic, prognostic, and treatment-monitoring tools.

Among the most well-documented markers of neuroinflammation in long COVID are the cytokines IL-6, TNF-α, and CCL2/MCP-1. Elevated levels of these molecules have been consistently found in the serum and CSF of long COVID patients, particularly those reporting cognitive dysfunction, fatigue, and sensory disturbances. IL-6 plays a central role in promoting glial activation and increasing BBB permeability, thereby facilitating immune cell infiltration into the CNS [16]. TNF-α contributes to synaptic disruption and neuronal death through excitotoxic pathways, while CCL2 is instrumental in recruiting monocytes and other immune cells across the BBB, sustaining chronic inflammation [115,116]. A recent longitudinal study by Tilikete et al. [117] showed that elevations in IL-6 and TNF-α levels persisted for up to a year after acute infection and were significantly associated with reduced cognitive performance and increased fatigue severity. More recently, attention has turned to CXCL13, a B-cell-attracting chemokine, as a potential novel marker of chronic intrathecal inflammation in long COVID, especially in patients with encephalopathy and cognitive slowing [118]. This chemokine, commonly studied in multiple sclerosis and Lyme neuroborreliosis, may offer additional insights into B-cell-mediated mechanisms in post-COVID brain dysfunction [119].

Beyond inflammatory mediators, evidence of structural neural damage in long COVID is supported by elevated serum concentrations of NFL and GFAP. NFL, a cytoskeletal protein released during axonal injury, has been repeatedly found to be increased in individuals with long COVID, memory loss, brain fog, and peripheral neuropathy [23,120,121]. A key study by Peluso et al. [122] demonstrated that serum levels of both NfL and GFAP markers of axonal and astrocytic injury were significantly elevated in individuals with PASC and positively correlated with pro-inflammatory cytokines such as IL-6 and TNF-α. This suggests that persistent immune activation may directly contribute to ongoing CNS injury. Frontera et al. [123] further confirmed that these biomarkers remain elevated in patients with neurological manifestations of COVID-19, reinforcing concerns about chronic neuroinflammatory processes. Additionally, a systematic review by Huang et al. [120] cited longitudinal data indicating that NfL and GFAP levels may remain abnormally high for more than 12 to 18 months in a subset of patients. These findings raise the possibility of a smoldering neurodegenerative process in vulnerable individuals. Reviews such as Greene et al. [124] support these observations, highlighting the role of cytokine-driven BBB disruption and sustained neuroinflammation in long COVID.

Furthermore, autoimmune and dysautonomic mechanisms have also emerged as important contributors to long COVID-associated neurological dysfunction. Some patients exhibit autoantibodies targeting gangliosides, which are glycolipids that are highly expressed on peripheral nerves [125]. These antibodies are characteristic of post-infectious GBS and have been identified in individuals with sensory loss and muscle weakness following COVID-19 [97]. Others present with antibodies targeting adrenergic or muscarinic receptors, which are often associated with POTS and autonomic dysfunction [126]. Such autoantibodies likely reflect a form of molecular mimicry, whereby immune responses directed against SARS-CoV-2 cross-react with host nervous tissue. In parallel, immunophenotyping studies have revealed signs of T-cell exhaustion, characterized by persistent expression of inhibitory receptors such as programmed cell death protein 1 (PD-1) and T-cell immunoglobulin and mucin-domain containing-3 (TIM-3) [127]. Chronic T-cell activation and dysregulated B-cell maturation, including expansions of double-negative B cells and plasmablasts, suggest that some long COVID patients develop a sustained autoimmune or autoinflammatory state even in the absence of prior autoimmune disease [128].

Biomarker findings are increasingly being complemented by advanced neurodiagnostic imaging and electrophysiological techniques, offering a multimodal view of post-COVID neuropathology. Neuroimaging studies using magnetic resonance imaging (MRI) and PET have identified hypometabolism in the frontal and parietal cortices of patients with cognitive impairment, along with white matter microstructural changes suggestive of axonal injury [20]. Electroencephalography (EEG) has revealed diffuse cortical slowing, frontal intermittent rhythmic delta activity (FIRDA), and abnormal waveforms in individuals with long COVID encephalopathy [129,130,131]. More recently, functional near-infrared spectroscopy (fNIRS) has been employed to assess brain perfusion in real time, identifying impaired hemodynamic responses during cognitive tasks in long COVID patients with brain fog [129,132]. These non-invasive tools not only help validate the presence of CNS dysfunction but also contextualize biomarker elevations within a neuroanatomical and functional framework.

The future of long COVID diagnosis likely lies in the integration of these biomarkers into clinical workflows, enabling more personalized and timely care. Serial measurements of IL-6, TNF-α, GFAP, and NFL may help track disease progression, assess response to immunomodulatory treatments such as corticosteroids or biologics, and guide decisions around rehabilitation and cognitive therapy. In addition, point-of-care cytokine assays and dried blood spot testing may bring biomarker testing into primary care settings, addressing a major bottleneck in the early identification of long COVID patients with neuroimmune involvement [133]. Ultimately, combining biomarker data with imaging and clinical phenotyping will facilitate more precise classification of long COVID subtypes and inform future trials targeting the immune–CNS interface.

**Figure 2:**
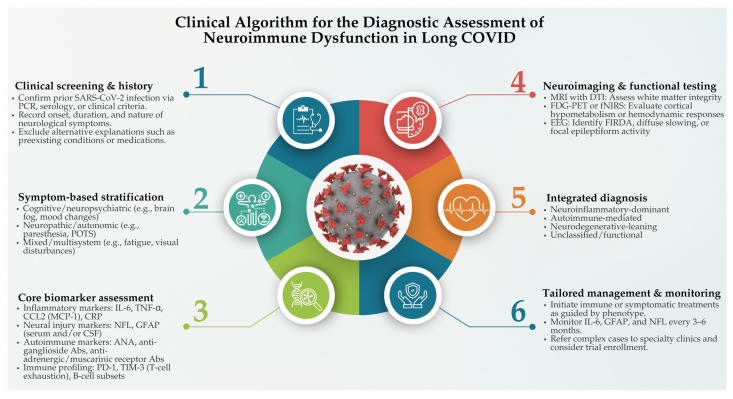
Clinical algorithm for the diagnostic assessment of neuroimmune dysfunction in long COVID. This algorithm provides a six-step framework for evaluating suspected neuroimmune dysfunction in individuals with long COVID. Clinical screening and history include confirmation of prior SARS-CoV-2 infection and documentation of neurological symptoms while ruling out alternative etiologies. Symptom-based stratification categorizes patients into cognitive/neuropsychiatric, neuropathic/autonomic, or mixed symptom clusters. Core biomarker assessment encompasses inflammatory markers, e.g., interleukins-6 (IL-6) and tumor necrosis factor-alpha (TNF-α); neural injury markers, e.g., glial fibrillary acidic protein (GFAP) and neurofilament light chain (NFL); auto-antibodies; and immune cell profiling. Neuroimaging and functional testing involve magnetic resonance imaging (MRI) with diffusion tensor imaging (DTI), fludeoxyglucose-positron emission tomography (FDG-PET) or functional near-infrared spectroscopy (fNIRS), and electroencephalography (EEG) to assess white matter changes, cortical metabolism, and electrical activity. Integrated diagnosis considers whether the phenotype is neuroinflammatory, autoimmune-mediated, neurodegenerative-leaning, or functional. Tailored management and monitoring recommend phenotype-guided therapy, serial biomarker monitoring (every 3–6 months), and referral of complex cases for specialty evaluations or clinical trial enrollment.

To address the diagnostic complexity of neuroimmune manifestations in long COVID, a structured clinical algorithm is essential. Figure 2 outlines a proposed stepwise framework for the diagnostic assessment of neuroimmune dysfunction in affected individuals.

## 6. Risk Factors for Neuro-COVID: Immune Vulnerability and Susceptibility Determinants

The heterogeneity of long COVID, particularly in its neurological manifestations, underscores the complex interplay between viral pathogenesis and host susceptibility. While SARS-CoV-2 has demonstrated neurotropic and immunomodulatory properties, not all individuals exposed to the virus develop neuro-COVID. This suggests that specific host-related factors, ranging from immune aging and hormonal regulation to genetic polymorphisms and metabolic comorbidities, modulate vulnerability to long-term neuroimmune dysfunction. Dissecting these factors is crucial for identifying at-risk individuals and for informing precision-based therapeutic strategies.

Aging remains one of the most consistent risk factors for neuro-COVID, largely due to immunosenescence, i.e., a state marked by diminished adaptive immune function, reduced naïve T-cell output, and increased memory cell accumulation [134]. This is often accompanied by “inflammaging”, a pro-inflammatory baseline driven by persistent activation of innate immune pathways, particularly NF-κB and NLRP3 inflammasomes [135]. Older individuals also exhibit impaired type I interferon responses and reduced antigen presentation capacity, leading to delayed viral clearance [136]. Critically, aging is associated with compromised BBB integrity due to endothelial senescence and oxidative stress, facilitating peripheral immune cell infiltration into the CNS. Studies have shown that aged COVID-19 patients have prolonged cerebrovascular inflammation and increased pericyte damage, contributing to cognitive impairment and neurovascular uncoupling [137].

On the other hand, the paradoxical pattern of long COVID, where women were more frequently reporting chronic symptoms despite lower acute mortality, points to a sexually dimorphic immune response [138,139]. Estrogen enhances T-helper 1 (Th1) responses and promotes robust B-cell function, which may increase resistance to acute infection but heighten the risk of post-infectious autoimmunity [140,141]. Conversely, testosterone dampens innate immunity and may reduce the intensity of cytokine storms but also contributes to prolonged viral persistence in tissues [142]. Beyond hormones, sex-linked genes on the X chromosome, including toll-like receptor 7 (TLR7), a key sensor for viral RNA, may provide females with an immunological advantage in pathogen detection, albeit at the cost of increased autoimmune susceptibility [143]. Emerging data from longitudinal studies show that females with long COVID demonstrate higher levels of autoantibodies (e.g., anti-IFN-α and anti-phospholipid), especially in those with persistent fatigue and cognitive dysfunction, suggesting that estrogen-enhanced immunity may become maladaptive in the post-viral phase [144].

In addition, patients with autoimmune diseases such as SLE, rheumatoid arthritis, or multiple sclerosis are at a heightened risk of neuro-COVID due to their preexisting immune dysregulation. These individuals often have enhanced antigen-presenting cell activity and a lower threshold for T- and B-cell activation [91,92,93]. Upon SARS-CoV-2 infection, these hyperactive immune networks can cause misdirected responses toward self-antigens through molecular mimicry. For instance, recent proteomic studies have shown homology between the SARS-CoV-2 spike protein and the myelin basic protein, potentially triggering demyelinating processes [30]. Clinical reports document new-onset autoimmune encephalitis and GBS in such patients post-COVID, often with elevated CSF cytokines and intrathecal IgG synthesis [145].

In terms of genetic susceptibility, genomic insights are beginning to uncover why some individuals are predisposed to persistent neurological symptoms. Certain HLA alleles, such as HLA-B15:01 and DRB104:01, have been associated with heightened CD8+ T-cell responses, potentially skewing toward autoimmunity [146,147]. Polymorphisms in the *IFNAR2*, *OAS1*, and *IL-6* genes correlate with more severe systemic inflammation and poor neurological recovery [148,149]. Novel research using multi-omics approaches has revealed that individuals with persistent neuro-COVID symptoms often have upregulated expression of genes involved in mitochondrial dysfunction and oxidative phosphorylation, especially in CNS-resident immune cells [150]. These findings suggest that inherited differences in immune metabolism and neuroimmune crosstalk can influence the trajectory of post-viral neurological sequelae.

Apart from genetics, metabolic syndrome and cardiovascular comorbidities are also significant risk factors. Obesity, type 2 diabetes, and hypertension create a systemic environment of low-grade inflammation, characterized by elevated levels of leptin, CRP, and IL-6. These comorbidities predispose individuals to a more intense cytokine response during acute infection and hinder the resolution of inflammation thereafter. Adipose tissue, a source of pro-inflammatory cytokines and a reservoir for viral RNA, may prolong immune activation [151]. In COVID-19 patients with metabolic syndrome, brain imaging studies show increased perivascular inflammation and impaired neurovascular coupling, correlating with cognitive fatigue [152]. Vascular risk factors also contribute to endothelial damage and BBB permeability, facilitating CNS exposure to peripheral cytokines and autoantibodies [11].

Finally, persistent viral reservoirs, i.e., a hidden driver of chronic immune stimulation, are also crucial. Persistent SARS-CoV-2 RNA and proteins have been detected in gut biopsies, lymphoid tissue, and even the CNS months after acute infection resolution [153]. This antigenic persistence may drive chronic immune stimulation via memory T cells and tissue-resident macrophages [154]. Notably, studies using spatial transcriptomics of the brain post-COVID have revealed localized expression of viral nucleocapsid proteins in astrocytes and perivascular macrophages, co-localizing with upregulated ISGs and complement genes [155,156]. These findings support the concept that incomplete viral clearance, possibly due to suboptimal interferon responses or immunoprivileged niche seeding, sustains glial activation, cytokine production, and neurodegeneration.

In summary, the risk of neuro-COVID is not evenly distributed across populations. It is shaped by a convergence of biological factors, including age, sex, genetics, immune history, and comorbid conditions. What distinguishes neuro-COVID from typical post-viral syndromes is the apparent entrenchment of immune dysregulation within CNS compartments, often driven by both systemic and localized factors. Identifying high-risk individuals through immunogenetic screening and biomarker profiling may enable early intervention strategies to dampen aberrant immune responses and preserve neurological function.

## 7. Limitations and Future Directions

Despite increasing recognition of long COVID as a neuroimmune condition, significant gaps remain in our understanding of its pathogenesis, diagnostics, and treatment. A key challenge in managing neuro-COVID lies in the heterogeneity of clinical phenotypes and underlying immune responses, particularly in patients with comorbidities such as diabetes, cardiovascular disease, or neurodegenerative conditions. While broad-spectrum anti-inflammatory treatments (e.g., corticosteroids and tocilizumab) have shown some benefit, they may not be effective or safe in all populations [157].

Moreover, current studies are often limited by small sample sizes, a lack of standardized diagnostic criteria, and inconsistent follow-up durations, which complicate the identification of causative mechanisms and effective therapies. There is an urgent need for longitudinal cohort studies that integrate clinical, immunology, and neuroimaging data to map the natural history of neuro-COVID. Such studies should prioritize the personalized therapies tailored to patient-specific biomarker profiles such as IL-6, TNF-α, GFAP, and NFL to capture dynamic changes in immune and neural injury over time. Moreover, multi-omics approaches, including transcriptomics, proteomics, and single-cell immune profiling, could help elucidate the molecular pathways underpinning persistent inflammation and autoimmunity in the CNS.

The role of COVID-19 vaccines in preventing neurological complications remains under investigation, with some studies suggesting a reduced incidence of long COVID symptoms in vaccinated individuals. Another critical direction involves stratifying patients based on immunogenetic risk factors. Recent data from genome-wide association studies (GWAS) have identified HLA alleles (e.g., HLA-B15:01 and HLA-DRB104:01) and interferon signaling polymorphisms as potential predictors of long COVID severity and duration [158,159]. Understanding how these variants modulate neuroimmune responses could guide the development of predictive models and precision therapeutics.

Moreover, therapeutic research must also move beyond symptom management toward immune-directed treatments. Novel therapeutic approaches, including monoclonal antibodies, small molecule inhibitors (e.g., JAK/STAT blockers), and agents targeting glial reactivity or mitochondrial function, are being explored [160,161]. While small observational studies suggest benefits of immunomodulatory agents like corticosteroids [162], IVIG, and low-dose naltrexone [163], randomized controlled trials remain scarce. Importantly, the optimal timing for intervention, whether during the acute phase, early recovery, or chronic stage, has yet to be established. Additionally, integrating omics-based diagnostics, immune phenotyping, and AI-driven stratification could facilitate this transition toward precision neuro-COVID care. Future clinical trials should be designed to evaluate both efficacy and safety across diverse patient populations, ideally guided by biomarker profiles and phenotypic subtypes.

Moreover, as a complementary approach to immunological and molecular profiling, artificial intelligence (AI) and machine learning (ML) techniques are increasingly being employed to stratify long COVID patients based on risk, symptom clusters, and immune signatures. These tools enable the integration of complex datasets, including transcriptomics, neuroimaging, biomarker profiles, and clinical features, to identify distinct neuroimmune phenotypes. For instance, unsupervised learning algorithms have been used to classify long COVID into endotypes characterized by dominant inflammatory, neurodegenerative, or autoimmune features [164]. Such precision-based stratification holds promise for tailoring therapeutic interventions and predicting clinical trajectories.

In addition, emerging SARS-CoV-2 variants continue to pose a threat, with preliminary evidence suggesting that viral mutations may differentially influence neurotropism and immune escape [165]. As such, variant-specific immune characterization will be necessary to understand their potential for neuroinvasion or prolonged antigenic persistence in the CNS or immune-privileged sites. Finally, interdisciplinary research linking immunology, neurology, virology, and data science is essential to advance the field. Developing integrated diagnostic frameworks that combine biomarkers, functional imaging, EEG, and cognitive assessments will be critical in creating actionable clinical pathways and informing treatment algorithms.

## 8. Conclusions

The neurological sequelae of long COVID represent a critical frontier in post-pandemic medicine. Mounting evidence indicates that these manifestations are not merely residual effects of viral infection but are driven by complex, persistent disruptions in immune regulation. SARS-CoV-2 acts as a powerful immunomodulatory agent that is capable of triggering chronic neuroinflammation, autoimmunity, and glial activation, even after viral clearance. Understanding long COVID as a neuroimmune disorder reframes how we diagnose, treat, and monitor affected individuals. Rather than a one-size-fits-all model, what is needed is a precision approach guided by immune and neural biomarkers, patient-specific risk factors, and longitudinal data. Immune-targeted therapies hold significant promise but require careful validation through well-designed clinical trials and biomarker-informed stratification. As we continue to uncover how SARS-CoV-2 reshapes the immune–nervous system axis, these insights will not only improve outcomes for those with long COVID but also deepen our understanding of post-viral neuroimmune syndromes more broadly. Addressing this challenge demands an interdisciplinary effort that unites immunology, neurology, virology, and systems biology to develop effective, individualized solutions for one of the most enduring legacies of the COVID-19 pandemic.

## Figures and Tables

**Table 1:** Immune biomarkers associated with post-COVID-19 neurological syndromes.

Condition	Neurological Symptoms	Immune Biomarkers
Cognitive dysfunction	Brain fog and memory loss	IL-6, GFAP, NFL, and TNF-α
Peripheral neuropathy	Numbness and tingling	IL-8, TNF-α, and autoantibodies
Depression and anxiety	Sleep disturbance and fatigue	IL-1β, IFN-γ, and CRP
Postural orthostatic tachycardia syndrome (POTS)	Tachycardia and orthostatic intolerance	IL-6, MCP-1, and CD30
Parkinsonism	Tremors and rigidity	α-synuclein, IL-1β, and sTNFRs
Guillain–Barré syndrome (GBS)	Muscle weakness and paralysis	Anti-ganglioside antibodies and IL-12

IL, interleukin; GFAP, glial fibrillary acidic protein; NFL, neurofilament light chain; TNF-α, tumor necrosis factor alpha; IFN-γ, interferon gamma; CRP, C-reactive protein; MCP-1, monocyte chemoattractant protein-1; CD30, tumor necrosis factor receptor superfamily member 8; and sTNFRs, soluble tumor necrosis factor receptors. The biomarkers listed are based on currently available studies and may vary across cohorts. This table is not exhaustive and highlights selected conditions with emerging evidence of immune-mediated mechanisms.

**Table 2:** Variant-specific neuropathogenesis of SARS-CoV-2.

Variant	Key Spike Mutations	Neuroinvasive Potential	Immune Signature	CNS Cell Tropism	Clinical Features
D614G [99,100,101,102]	D614G	High (olfactory and cortical)	CXCL10, IFN-β, and IL-6	Neurons and microglia	Anosmia, brain fog, and encephalitis
Delta [103,104,105,106,107]	L452R and T478K	Moderate–High	IL-1β and MCP-1	Astrocytes and pericytes	Headache and encephalopathy
Omicron (BA.1–XBB) [19,108,109,110]	Multiple S1/S2	Low direct and high indirect	IL-6, CXCL8, and HSP70	Astrocytes and pericytes	Cognitive dysfunction and fatigue
Alpha [109,111,112,113]	N501Y and P681H	Low	IL-12 and IFN-γ	Endothelial cells	Mild anosmia and fatigue
Beta [109,111,112,113]	E484K and K417N	High (olfactory and cortical)	IL-18 and TNF-α	Glial cells	Neurovascular symptoms

CNS, central nervous system; CXCL, C-X-C motif chemokine ligand; IFN, interferon; IL, interleukin; MCP-1, monocyte chemoattractant protein-1; HSP70, heat shock protein 70; TNF-α, tumor necrosis factor alpha. Tropism refers to the preferential targeting of specific CNS cell types by each variant, based on in vitro and in vivo models. Immune signatures reflect dominant cytokines/chemokines identified in cerebrospinal fluid (CSF), serum, or experimental models. Clinical features are illustrative and may vary depending on patient demographics and comorbidities.

## Abbreviations

The following abbreviations are used in this manuscript:

ACE2	Angiotensin-converting enzyme 2
ADEM	Acute disseminated encephalomyelitis
BBB	Blood–brain barrier
CCL2	C-C motif ligand 2
CNS	Central nervous system
CRP	C-reactive protein
CSF	Cerebrospinal fluid
CSVD	Cerebral small vessel disease
DTI	Diffusion tensor imaging
EEG	Electroencephalography
FDG-PET	Fludeoxyglucose-positron emission tomography
FIRDA	Frontal intermittent rhythmic delta activity
fNIRS	Functional near-infrared spectroscopy
GBS	Guillain–Barré syndrome
GFAP	Glial fibrillary acidic protein
HPA	Hypothalamic–pituitary–adrenal
HSP70	Heat shock protein 70
IFN-γ	Interferon-γ
IL	Interleukin
JAK/STAT	Janus kinase/signal transducer and activator of transcription
MCP-1	Monocytic chemotactic protein 1
MMP	Matrix metalloproteinases
MOG	Myelin oligodendrocyte glycoprotein
NFL	Neurofilament light chain
NF-κB	Nuclear factor kappa beta
NLRP3	NLR family pyrin domain containing 3
NMDA	N-methyl-D-aspartate
PASC	Post-acute sequelae of SARS-CoV-2
PET	Positron emission tomography
POTS	Postural orthostatic tachycardia syndrome
PTSD	Post-traumatic stress disorder
SARS-CoV-2	Severe Acute Respiratory Syndrome Coronavirus 2
SCFA	Short-chain fatty acids
SFN	Small fiber neuropathy
TLR7	Toll-like receptor 7
TMPRSS2	transmembrane protease, serine 2
TNF-α	Tumor necrosis factor-alpha
VEGF	Vascular endothelial growth factor
vWF	Von Willebrand factor
